# Effect of Squalane as a Carrier in O/W Nanoemulsions for Dermal Delivery of Vitamin E

**DOI:** 10.1021/acsomega.5c08561

**Published:** 2025-12-03

**Authors:** Aniely Cristina de Souza, Caroline Casagrande Sipoli, Rafael Block Samulewski, Ana Caroline Raimundini Aranha, Rafael Oliveira Defendi, Rúbia Michele Suzuki

**Affiliations:** † 74354 Universidade Tecnológica Federal do Paraná, Postgraduate Program in Chemical Engineering (PPGEQ-AP), Rua Marcilio Dias, 635, Apucarana, PR 86812-460, Brazil; ‡ Universidade Estadual de Maringá, Postgraduate Program in Chemical Engineering, Department of Chemical Engineering, Av. Colombo 5790, Bloco D90, Maringá, PR 87020-900, Brazil

## Abstract

Vitamin E, a potent antioxidant, is widely used in cosmetics to combat skin aging, but its lipophilic nature poses challenges for dermal delivery. Squalane, a biocompatible carrier, may enhance the stability and skin permeation of vitamin E in oil-in-water (O/W) nanoemulsions. O/W nanoemulsions containing vitamin E were formulated using high shear homogenization with varying coconut oil-to-squalane ratios (0:3, 1:2, 2:1, 3:0). Physicochemical properties (particle size, polydispersity index, ζ-potential, pH, conductivity) were characterized. In vitro release kinetics were assessed via dialysis, quantified by HPLC, and skin permeation was evaluated using Franz diffusion cells with porcine ear skin. Nanoemulsions exhibited hydrodynamic diameters of 107.67 ± 7.57 to 134.38 ± 3.15 nm, polydispersity indices of 0.21–0.24, and ζ-potentials of −37.4 ± 0.80 to −45.3 ± 0.90 mV, indicating high stability (*p* < 0.05, ANOVA). The encapsulation efficiency for vitamin E was >99% across all formulations. These properties were maintained over 90 days of storage at 25 ± 1 °C in the dark, with no significant changes in diameter, PDI, or ζ-potential (*p* > 0.05, ANOVA). Accelerated stability tests (centrifugation, thermal stress up to 80 °C) confirmed robustness, with minimal changes in key parameters. The V.E 0:3 formulation (pure squalane) showed the highest vitamin E release (0.341 h^–1^, Korsmeyer-Peppas model, *R* ^2^ = 0.993) and skin permeation (164.71 ± 10.06 μg cm^–2^ over 24 h, *p* < 0.05), significantly outperforming coconut oil-rich formulations (V.E 3:0:11.72 ± 12.41 μg cm^–2^). Steady-state flux (Jss) for V.E 0:3 was 35.1 ± 1.4 μg cm^–2^ h^–1^, compared to 0.1 ± 0.2 μg cm^–2^ h^–1^ for V.E 3:0 (*p* < 0.05). Squalane-based O/W nanoemulsions significantly enhance the stability and dermal delivery of vitamin E, with the V.E 0:3 formulation demonstrating superior release kinetics and skin permeation. These findings support the potential of squalane-based nanoemulsions for effective topical delivery in cosmetic applications, warranting further in vivo studies to confirm efficacy and safety.

## Graphical abstract

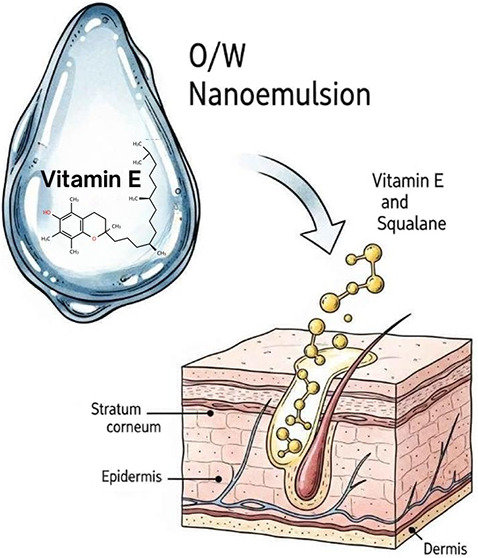

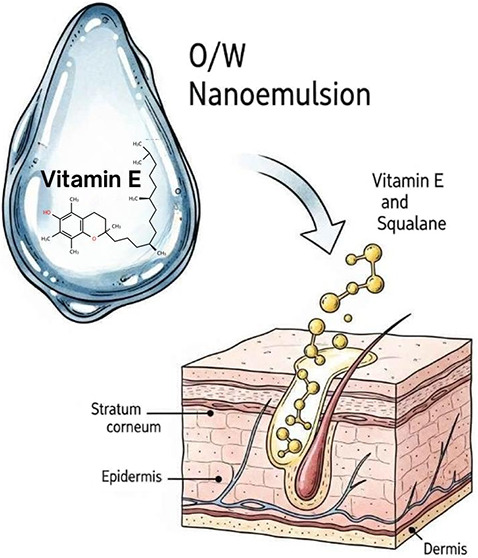

## 1 Introduction

The skin is the human body’s largest organ, with a surface area of approximately 25 m^2^. It plays a crucial role as the first line of defense against harmful external factors, including pollutants, radiation, and microorganisms.^1,2^ In addition to its protective functions, the skin is responsible for maintaining body temperature and preventing water loss.^1,3^ Furthermore, the appearance of the skin significantly influences self-esteem and social perception.^4,5^ This connection between skin health and self-image has driven the increased consumer demand for active ingredients that combat the visible effects of aging.

Aging is a multifactorial process influenced by both intrinsic and extrinsic factors, both of which contribute to biochemical changes in the skin. These changes include the excessive formation of reactive oxygen species (ROS), which damage functional structures and lead to morphological and physiological alterations in the skin.^6^ Intrinsic aging, which is largely genetically determined, is characterized by expression lines, sagging, and a thinner epidermis due to a decline in cell renewal and collagen synthesis. Extrinsic aging, on the other hand, is primarily caused by environmental factors such as UV radiation, pollution, and lifestyle habits. It is considered more detrimental, resulting in irregular pigmentation, reduced keratinocyte activity, and loss of skin elasticity, which occurs due to the fragmentation of collagen and elastin fibers in the dermal-epidermal junction.^7–9^

The visible effects of aging significantly impact self-esteem and social perception, driving consumer demand for cosmeceutical products that combine aesthetic enhancement with therapeutic benefits.^10,11^ The global cosmetics market, fueled by growing awareness of plant-derived bioactives and sustainable solutions, continues to expand rapidly, with social media and Internet platforms amplifying interest in antiaging formulations.^12,13^

Among the most recognized plant-derived antioxidants are phenolic compounds (including flavonoids and nonflavonoids), terpenoids (including carotenoids), and vitamins such as A, C, and E.^14,15^ Among these, vitamin E stands out due to its antioxidant, anti-inflammatory, and photoprotective properties. Discovered by Evans and Bishop in 1922, vitamin E consists of two main groups—tocopherols and tocotrienols, each with four isomers (α, β, γ, and delta). It is widely distributed in plant- and animal-derived oils, with long-known antioxidant properties that protect skin cells from oxidative stress.^16,17^ Additionally, vitamin E has demonstrated significant potential in preventing cancer and treating degenerative diseases, including Alzheimer’s and Parkinson’s disease, through mechanisms such as tumor growth modulation, prevention of cell proliferation, and induction of apoptosis.^18,19^

Another highly beneficial compound frequently utilized in the cosmetics industry is squalane, a saturated form of squalene. Squalene is a triterpene that is a key intermediate in the sterol biosynthetic pathway. It is found in a variety of plant oils, such as those derived from amaranth, olives, peanuts, soybeans, rice, wheat germ, and grape seeds, and is also present in human tissues, comprising approximately 12% of the lipid content of human skin.^20^ Due to its antioxidant properties, squalane is widely used in the pharmaceutical and cosmetic industries for its moisturizing and skin-penetrating capabilities.^21^ It is particularly known for improving skin appearance by reducing expression lines^22^ and enhancing the delivery of other active compounds.^22,23^

Squalane-based delivery systems, such as nanoemulsions and nanostructured lipid carriers, have been explored for delivering actives like retinol and coenzyme Q10, demonstrating enhanced skin retention and stability compared to traditional emulsions.^20–23^ For example, Oliveira et al. showed that squalane in W/O emulsions increased polyphenol retention in the skin by extending residence time, reducing transdermal flux.^21^ In contrast, other penetration enhancers like oleic acid and propylene glycol often promote deeper transdermal penetration, which may lead to systemic absorption undesirable for cosmetic applications.^20–23^ Squalane’s ability to enhance localized skin retention makes it particularly suitable for vitamin E delivery, where stratum corneum accumulation is preferred. Coconut oil, rich in medium-chain fatty acids like lauric acid (approximately 50% of its composition), was selected as a complementary oil phase due to its antibacterial properties against pathogens like *Propionibacterium acnes* and its ability to strengthen the skin barrier by reducing transepidermal water loss. This combination of squalane’s penetration-enhancing capabilities and coconut oil’s therapeutic benefits offers a synergistic approach to optimizing vitamin E delivery, which remains underexplored in the literature.^20–23^

Despite the broad range of applications, one of the major challenges in the cosmetic industry, especially when working with lipophilic active ingredients, is the difficulty in incorporating these compounds into water-based systems. This limitation has been addressed through the use of nanotechnology, which enables the development of novel delivery systems that improve the solubility, stability, and efficacy of active ingredients.^24^ Nanotechnology allows the creation of nanoemulsions, which are colloidal systems with droplets in the nanometer range that offer enhanced solubility and bioavailability for lipophilic substances, facilitating their incorporation into aqueous-based formulations. Nanoemulsions, which can be either oil-in-water (O/W), water-in-oil (W/O), or multiple emulsions (W/O/W and O/W/O), can be used in a variety of applications, including drug delivery, nutrient encapsulation, and cosmetic formulation.^25,26^

The application of nanoemulsions in cosmetics has gained considerable interest due to their ability to improve the absorption and retention of active ingredients in the stratum corneum, the outermost layer of the skin. By enhancing the dispersion of lipophilic compounds in aqueous systems, nanoemulsions not only improve the stability of the actives but also ensure their more efficient delivery to the skin, thus maximizing their therapeutic and cosmetic effects.^27,28^

Several studies have explored the use of nanoemulsions for delivering vitamin E in cosmetic and pharmaceutical applications, demonstrating their potential to enhance the bioavailability and skin penetration. For instance, Kong et al.^29^ developed oil-in-water nanoemulsions using vitamin E as the active ingredient, hyaluronic acid as the aqueous phase, and a surfactant system composed of Tween 80 and Span 20, reporting improved skin retention but limited transdermal permeation (approximately 8.29 μg mL^–1^ over 24 h). Similarly, Schreiner et al.^30^ formulated a nanocream stabilized with saponins and containing 1% vitamin E, achieving skin retentions of approximately 11–21 μg cm^–2^, depending on the formulation complexity. Other studies have investigated nanoemulsions with different oil phases, such as medium-chain triglycerides or essential oils, to deliver vitamin E, often focusing on stability and antioxidant activity but with varying degrees of skin permeation efficiency.^17^ In contrast, the present study focuses on the use of squalane, a biocompatible and skin-friendly carrier, in combination with coconut oil in varying ratios to formulate oil-in-water nanoemulsions. This approach aims to optimize both the stability and skin permeation of vitamin E, leveraging squalane’s unique moisturizing and penetration-enhancing properties to improve delivery efficiency compared to previous systems. Unlike prior studies, our work systematically investigates the impact of the coconut oil-to-squalane ratio on nanoemulsion characteristics and performance, providing insights into the role of lipid composition in enhancing vitamin E delivery for topical applications.

While squalane is a well-established component in cosmetic formulations due to its moisturizing and penetration-enhancing properties, its combination with coconut oil in the O/W nanoemulsions for vitamin E delivery remains underexplored. This study introduces a novel approach by systematically investigating the impact of varying coconut oil-to-squalane ratios (0:3, 1:2, 2:1, and 3:0) on the physicochemical properties, stability, and performance of O/W nanoemulsions. Coconut oil, rich in medium-chain fatty acids like lauric acid, offers unique benefits such as antibacterial activity and enhanced skin barrier function, which complement squalane’s penetration-enhancing capabilities. Unlike previous studies that focus on single oil phases or different active compounds, this work provides a comprehensive analysis of how the synergy between coconut oil and squalane modulates droplet size, ζ-potential, release kinetics, and skin permeation of vitamin E. This systematic approach to combining squalane and coconut oil represents a significant advancement in designing nanoemulsion-based delivery systems for lipophilic actives, offering the potential for enhanced efficacy in cosmetic and dermatological applications.

The development of nanoemulsion-based delivery systems for the codelivery of squalane and vitamin E presents a promising approach to addressing the challenges of formulating effective cosmetic products. These systems not only enhance the stability and bioavailability of the bioactive ingredients but also offer the potential for improved skin penetration, increased efficacy, and sustained release of the active compounds. The goal of this study is to investigate the formulation of an O/W nanoemulsion containing squalane and vitamin E, focusing on its physicochemical characterization, stability, and in vitro performance using the Franz cell model to evaluate its potential for topical application.

## 2 Materials and Methods

### 2.1 Materials

Dialysis bags (Viskase-, model MD 34, cutoff molecular mass 3500 Da), Nylon syringe filters (0.22 μm), Franz diffusion cell system, parafilm, soy lecithin (Solec), coconut oil (Copra), squalane (Sigma-Aldrich), polysorbate-Tween 80 (Chemical Dynamics), vitamin E (Essence Engineering), methanol UV/HPLC (Chemical Dynamics), acetonitrile (Sigma-Aldrich), ultrapure water, ethanol, and water solution (50%, v/v), KCl solution (0.1 mol L^–1^).

### 2.2 Methodology

#### 2.2.1 Characterization of Coconut Oil

##### 2.2.1.1 Acidity Index

The acidity index was determined according to the methodology of Adolfo Lutz^31^ with modifications. A mixture of ether and ethyl alcohol (2:1, v/v) was prepared along with a standardized potassium hydroxide solution (0.108 mol L^–1^). The procedure involved weighing three samples of coconut oil in 250 mL Erlenmeyer flasks, to which 25 mL of the ether-alcohol solution and two drops of phenolphthalein indicator were added. The acidity index (mg g^–1^) and the acidity percentage of the samples were calculated by using eqs 1 and 2, respectively.

1 $$acidity\;index\;\left(AI\right)\left(\frac{mg\;KOH}{g\;of\;sample}\right)=\frac{vCf56.1}{m}$$

2 $$\mathrm{acidity}\left((\%\left(g\;100\;g^{-1}\right)\mathrm{of}\;\mathrm{oleic}\;\mathrm{acid}\;C18:2)\right)=\left(\frac{\mathrm{vCf}282}{m}\right)$$

where *v* represents the volume of potassium hydroxide used in the titration (mL), *f* is the experimentally determined correction factor of the potassium hydroxide solution, *C* is the concentration of the potassium hydroxide solution (mol L^–1^), and m is the mass of the sample (g).

##### 2.2.1.2 Identification and Quantification of Fatty Acids

Fatty acid methyl esters (FAMEs) were prepared following the method described by Hartman and Lago^32^ and adapted by Maia.^33^ The FAMEs were separated using a gas chromatograph coupled with a mass spectrometer (GC-MS), employing a Shimadzu MS 5977A system. Separation was achieved on an RT-2560 capillary column (100 m length, 0.25 mm internal diameter, and 0.20 μm film thickness). The oven temperature program commenced at 190 °C, held for 1 min, followed by a ramp at 2 °C min^–1^ to 200 °C, where it was maintained for 2 min.

The temperature was then increased to 230 °C at the same rate and maintained for 12 min. Solutions were analyzed in triplicate with a 1.0 μL injection volume in splits (split ratio 100:1), and helium was used as the carrier gas at a flow rate of 1.232 mL min^–1^.

Identification of the fatty acids was performed by comparing the retention times of the FAMEs with those of standard mixtures containing geometric isomers of linoleic and α-linolenic acids (Sigma), along with their equivalent chain length (ECL) values, as outlined by Visentainer and Franco.^34^ Quantification of the identified fatty acids was based on an internal standard. The internal standard solution was prepared at a concentration of 1.0 mg mL^–1^ in iso-octane and was added to the esterification container after weighing the sample.

Fatty acid quantification in the coconut oil was carried out using eq 3, as described by Visentainer and Franco:^34^

3 $$m_{x}=\frac{A_{x}M_{p}F_{CT}}{A_{p}M_{A}F_{CEA}}$$

where *M_x_* represents the mass of fatty acid in mg g of oil^–1^, *M*_p_ is the mass of the internal standard in mg, *M*_A_ is the mass of the sample in g, *A*_p_ is the peak area of the internal standard in the chromatogram, *A_x_* is the peak area of the fatty acid, *F*_CEA_ is the methyl ester correction factor for fatty acid, and *F*_CT_ is the theoretical fatty acid correction factor.

#### 2.2.2 Preparation of Nanoemulsions

To optimize agitation speed, blank nanoemulsions were prepared at 8000, 12,500, 15,000, and 18,000 rpm. The speed of 15,000 rpm was selected based on optimal size, PDI, and stability over 28 days (*p* > 0.05 for sizes between 12,500 and 18,000 rpm).

Vitamin E-loaded nanoemulsions were formulated by dispersing an oil phase into an aqueous phase under high shear homogenization at 15,000 rpm. The oil phase was composed of soy lecithin (Solec) at a concentration of 30.0 mg mL^–1^, vitamin E at 20.0 mg mL^–1^, and a mixture of coconut oil and squalane in varying mass ratios of 0:3, 1:2, 2:1, and 3:0 (w/w), as detailed in Table 1. The aqueous phase was prepared by dissolving Tween 80 at 30.0 mg mL^–1^ in ultrapure water, and the final formulation volume was adjusted to 100 mL.

**Table 1:** Composition of the Nanoemulsion Systems

**name of sample**	**soy lecithin** (g)	**tween 80** (g)	**vitamin E** (g)	**coconut oil** (g)	**squalane** (g)
V.E 0:3	3.000	3.000	2.000		3.000
V.E 1:2	3.000	3.000	2.000	1.000	2.000
V.E 2:1	3.000	3.000	2.000	2.000	1.000
V.E 3:0	3.000	3.000	2.000	3.000	

Both phases were heated separately before emulsification: the aqueous phase was heated to 70 ± 0.1 °C, while the oil phase was heated to approximately 50 °C, just enough to ensure complete solubilization of the lipid components. Subsequently, the oil phase was slowly added to the aqueous phase under continuous stirring at ambient temperature (25 ± 0.1 °C) using a high shear homogenizer (Ultra-Turrax T-25, IKA, Germany). Homogenization was conducted for 15 min at 15,000 rpm, as optimized in preliminary experiments.

After homogenization, the nanoemulsions were allowed to equilibrate at room temperature (25 ± 0.1 °C) for 24 h before physicochemical characterization.

#### 2.2.3 Encapsulation Efficiency

Encapsulation efficiency (EE) was determined by ultrafiltration–centrifugation. Samples were centrifuged at 5000 rpm for 30 min using ultrafiltration tubes (cutoff 10 kDa). The filtrate was analyzed by HPLC for free vitamin E. EE was calculated as

4 $$EE\;\left(\%\right)=\frac{\left(total\;vita\min\;E-free\;vita\min\;E\right)}{total\;vita\min\;E}\times 100\%$$

#### 2.2.4 Determination of Kinetic Stability Parameters of the System

The samples were stored in the dark at a temperature of 25 ± 1 °C and analyzed 24 h post-preparation. Stability was further monitored over 90 days under the same conditions, with measurements of particle size, PDI, and ζ-potential taken at days 1, 7, 14, 28, 60, and 90. Accelerated stability was assessed via centrifugation (3000 rpm for 30 min) and thermal stress (heating from 4 to 80 °C in increments, holding 30 min at each temperature).

Particle size, polydispersity index (PDI), and ζ-potential were evaluated using dynamic light scattering (DLS) on a Litesizer 500 (Anton Paar) at 25 ± 1 °C. Before each measurement, the samples were diluted in a 1:50 ratio. Data analysis and graphing were performed using the OriginLab 2024 software.

Electrical conductivity was measured both in the blank nanoemulsions and in the systems containing the vitamins. A conductivity meter, calibrated with a 0.1 mol L^–1^ KCl solution, was employed at 25 ± 0.1 °C, with the electrode immersed directly into the solutions. pH values of the nanoemulsions were recorded using a calibrated pH meter (Bel Phs 3bw) at 25 ± 0.1 °C, with direct insertion of the electrode into the solutions.

#### 2.2.5 Morphological Analysis

The morphological analysis of the samples was carried out using scanning electron microscopy (SEM), following the methodology described by Divsalar et al.,^35^ with adaptations. Initially, the samples were dropped directly onto the sample holders (stubs) and dried using a lyophilizer.

After drying, the samples were sputter-coated with a thin layer of gold (99.99% purity) by using a Deton Vacuum V coater at 30 A for 40 s.

The analysis was performed by using a Tescan Vega LMU scanning electron microscope equipped with a secondary electron detector. Micrographs were acquired at magnifications of 5 and 10.0 kx to evaluate surface morphology.

#### 2.2.6 Vitamin E Release Kinetics via Dialysis

Following the methodology adapted from Sharipova et al.,^36^ the release profile of vitamin E was investigated in a 50% ethanol–water solution. A 4 mL volume of nanoemulsions was placed in dialysis bags (Viskase, model MD 34, molecular weight cutoff of 3500 Da) and immersed in 200 mL of the ethanol solution. The system was maintained under continuous stirring on a magnetic stirrer at room temperature. At predetermined time intervals (5, 10, 15, 30, 60, 120, 180, 360, 480, 720, and 1440 min), 3 mL aliquots were withdrawn from the medium and replaced with an equal volume of the ethanol–water solution (50%, v/v). Ethanol was included to enhance the solubility of vitamin E in the aqueous phase.

The samples were stored in the dark under refrigeration, and vitamin E concentrations were quantified using High-Performance Liquid Chromatography (HPLC-UV–vis, PerkinElmer Flexar) with a C18 Nucleosil 100–5 reversed-phase column. The mobile phase consisted of a mixture of acetonitrile/methanol (95:5, v/v). The analysis was performed at room temperature with a flow rate of 1.0 mL/min and UV detection at 297 nm. A calibration curve was constructed using standard vitamin E solutions in the concentration range of 0.00568 to 287.37 μg/mL in acetonitrile/methanol (95:5, v/v), yielding a linear regression equation of *y* = 1465.734*x* + 4002.248 (*R* ^2^ = 0.998). Before analysis, all samples were filtered through a 0.22 μm nylon syringe filter.

The release kinetics were evaluated using mathematical models described by Azhar et al.,^37^ Dash et al.,^38^ and Siepmann & Peppas^39^ with MATLAB software. The models used were: Zero-order (cumulative amount of vitamin released versus time, eq 5), First-order (cumulative logarithmic amount of vitamin versus time, eq 6), Higuchi^40^ (cumulative amount of vitamin released versus the square root of time, eq 7), and Korsmeyer-Peppas^41^ (logarithmic cumulative amount of vitamin released versus logarithmic time, eq 8).

5 $$M_{t}=M_{0}+K_{0}t$$

6 $$\log M_{t}=\log M_{0}+K_{1}t/2.303$$

7 $$M_{t}=k_{H}\sqrt{t}$$

8 $$\frac{M_{t}}{M_{\infty }}=K_{\mathrm{kp}}t^{n}$$

where *M*_0_ is the initial amount of vitamin in the medium, *M*_*t*_ is the amount of vitamin released at time *t*, *M*_∞_ is the amount of vitamin released at time ∞ (last point of the kinetics), *K*_0_, *K*_1_, *k*_H_, and *K*_kp_ are release rate constants, *n* is the release exponent, and *t* is the time.

#### 2.2.7 Skin Permeation Studies

An in vitro skin permeation assay was performed using full-thickness porcine ear skin as the model membrane and Franz diffusion cells, following the methodology described by Demisli et al.^42^ and Schreiner et al.,^30^ with adaptations. Ears were obtained from a freshly slaughtered adult pig (72 kg) sourced from a licensed facility. Ethical approval was not required as the animal was not specifically slaughtered for this study.

Franz diffusion cells with an effective diffusion area of 1.00 cm^2^, a receptor chamber volume of 11 mL, and a donor chamber volume of 5 mL were utilized. The ears were cleaned, dried, wrapped in aluminum foil, and frozen until required. The temperature of the water bath connected to the Franz diffusion cells was maintained at 32 ± 1 °C, simulating an average skin temperature. The receptor chambers were filled with a preheated mixture of water and ethanol (50%, v/v) and continuously stirred using a magnetic stirrer. The skin was placed between the donor and receptor chambers and allowed to equilibrate for 30 min. Subsequently, 4 mL of each formulation was added to the donor compartment and sealed with a Parafilm to prevent evaporation.

The release study was conducted over 24 h. At time intervals of 1, 2, 3, 4, 5, 6, 7, 8, and 24 h, 600 μL aliquots were withdrawn from the receptor chambers and replaced with the same volume of fresh, preheated medium. After completion of each experiment, the skin was excised, cut into small pieces, immersed in 10 mL of methanol, and subjected to ultrasonic extraction for 30 min to recover the retained vitamin.

The samples were stored in the dark under refrigeration, and vitamin E concentrations were quantified by high-performance liquid chromatography (HPLC-UV–vis, PerkinElmer Flexar) using a Nucleosil 100–5 reversed-phase C18 column. The mobile phase was a mixture of acetonitrile/methanol (95:5, v/v). The analysis was performed at room temperature with a flow rate of 1.0 mL/min and UV detection at 297 nm. A calibration curve (*y* = 1465.734*x* + 4002.248, *R* ^2^ = 0.998) was constructed using standard vitamin E solutions in the concentration range of 0.00568 to 287.37 μg/mL in acetonitrile/methanol (95:5, v/v). Before analysis, all samples were filtered through a 0.22 μm nylon syringe filter.

For control samples, vitamin E was dispersed in sweet almond oil at the same concentration as in the nanoemulsion samples, as described by Schreiner et al.^30^ Sweet almond oil was selected as the control due to its widespread use as a carrier for lipophilic actives like vitamin E in cosmetic formulations, providing a simple, lipid-based system that allows direct comparison with the nanoemulsions’ oil phase without the confounding effects of additional emulsifiers or stabilizers found in conventional creams or emulsions.^30^ This choice enables evaluation of the nanoemulsions’ enhanced delivery efficiency relative to a basic oil-based delivery system commonly used in cosmetics. However, we acknowledge that a conventional cream or emulsion containing vitamin E could provide a more clinically relevant comparison for topical applications. Future studies will incorporate such formulations as additional controls to better assess the nanoemulsions’ performance against standard cosmetic products. The release study was conducted as described above with the same sampling protocol and sample extraction procedure.

The cumulative penetration of vitamin E (*Q_t_*, μg cm^–2^) through the skin for both nanoemulsion and control samples was calculated using the equation described by Sintov and Shapiro^43^ (eq 9):

9 $$\mathrm{Qt}=V_{r}C_{t}+\sum_{i=0}^{t-1}V_{s}C_{i}$$

where *C_t_* is the drug concentration in the receptor solution at each sampling time, *C_i_* is the drug concentration of the *i*-th sample, and *V*_r_ and *V*_s_ are the volumes of the receptor solution and the sample, respectively. Based on the results, the total amount of vitamin E in the receptor compartment (μg cm^–2^), the amount of vitamin E retained in the skin (μg cm^–2^), and the percentage of vitamin E released during the experiment (%) were determined. The steady-state flux (Jss) was calculated by linear regression interpolation of the experimental data under steady-state conditions, according to the equation described by Kong et al.^29^ (eq 10).

10 $$\mathrm{Jss}=\frac{\left(\Delta \mathrm{Qt}\right)}{\left(\Delta \mathrm{tS}\right)}$$

In this equation, Jss represents the steady-state flux, Δ*Q_t_* is the change in the cumulative penetration of vitamin E, Δ*t* is the change in time, and *S* is the effective diffusion area of the skin (1.00 cm^2^). The apparent permeability coefficient (Kp, × 10^–3^ cm h^–1^) was calculated according to eq 11, also described by Kong et al.^29^

11 $$\mathrm{Kp}=\frac{\mathrm{Jss}}{C_{d}}$$

where *C*_d_ is the concentration of vitamin E in the donor compartment, and it was assumed that under sink conditions, the concentration of vitamin E in the receptor compartment is negligible compared to that in the donor compartment.

#### 2.2.8 Statistical Analysis

The data were subjected to analysis of variance (ANOVA) followed by Tukey’s post hoc test to assess significant differences, with a significance threshold set at *p* < 0.05. Statistical analyses were performed using RStudio software. Mathematical model fitting was conducted using MATLAB software.

## 3 Results and Discussion

### 3.1 Coconut Oil Characterization

#### 3.1.1 Acid Value

The coconut oil exhibited an acid value of 0.82 ± 0.01 mg of KOH g^–1^ and an oleic acid content of 0.04 ± 0.01%. According to the regulations set by ANVISA and the Codex Alimentarius–FAO/WHO, the maximum allowable acid value for cold-pressed, unrefined oils, such as coconut oil, is 4.0 mg of KOH g^–1^. These findings suggest that the coconut oil selected for the formulation of nanoemulsions complies with the established quality standards.

The acid value, defined as the amount of potassium hydroxide (KOH) (milligrams) required to neutralize one gram of oil, serves as a critical parameter for evaluating oil preservation. Factors such as hydrolysis, fermentation, and oxidation can lead to variations in hydrogen ion concentrations, resulting in the breakdown of triglycerides and the formation of free fatty acids, which are indicative of oil rancidity.^44^ Several variables, including the quality of the raw material and the conditions under which the oil is processed and stored, significantly impact the acidity of vegetable oils, underscoring the importance of this analysis in assessing oil quality and long-term stability.^45^

#### 3.1.2 Fatty Acid Identification and Quantification

The fatty acid profile of coconut oil is depicted in Supporting Information Figure S1, which outlines the lipid composition of this raw material.

The fatty acids present in the coconut oil were quantified, and the results are summarized in Supporting Information Table S1, expressed in milligrams per gram of total lipids (mg g^–1^), obtained through internal standardization.

The data reveal that lauric acid (C12:0) is the predominant fatty acid, by the standards set by ANVISA and the Codex Alimentarius–FAO/WHO. All quantified fatty acids were consistent with these established guidelines.

In addition to confirming the quality of the raw material, the presence of these fatty acids in cosmetic formulations may offer therapeutic benefits. The inclusion of oleic, lauric, and palmitic acids, for example, is known to exert antibacterial effects and could enhance the skin’s defense mechanisms against *P. acnes*.^46–48^

Linoleic acid, an essential fatty acid, is particularly significant for human skin health. Its high concentration in the stratum corneum helps prevent transepidermal water loss and plays a crucial role in maintaining the integrity of the epidermal cell membrane. Linoleic acid, along with linolenic and arachidonic acids, is essential for preventing skin disorders such as atopic dermatitis and psoriasis.^49^

Thus, the appropriate lipid composition in cosmetic formulations is essential for preserving the integrity and function of the skin barrier.^50^ A balanced profile of fatty acids can contribute to improved skin health by providing benefits such as antibacterial activity, enhanced hydration, and support for the skin’s natural defense mechanisms.^51^ Consequently, the careful selection of raw materials for the formulation of cosmetic and pharmaceutical products based on these fatty acids is critical for the prevention and treatment of various dermatological conditions.

### 3.2 Development of Nanoemulsions

The development of empty nanoemulsions was carried out with the oil phase maintained at a concentration of 3%. The oils were mixed in various ratios of coconut oil to squalane (3:0, 2:1, 1:2, and 0:3 g 100 mL^–1^), based on the determination of the optimal stirring speed. No phase separation was observed in any of the formulations, which qualitatively suggested the stability of the nanoemulsions. All emulsions exhibited a hydrodynamic mean diameter of less than 200 nm with a unimodal distribution (Figure 1).

**Figure 1:**
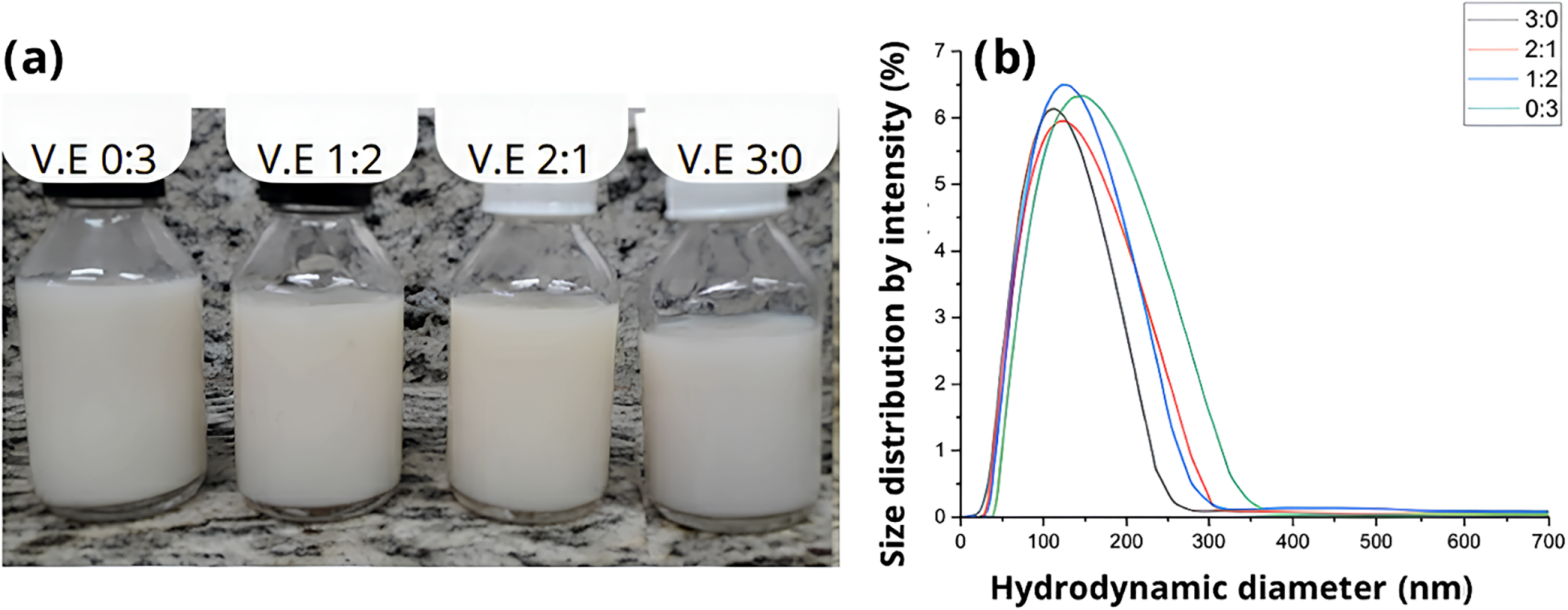
(a) Visual appearance of nanoemulsions developed with different proportions of coconut oil/squalane. (b) Size distribution.

The hydrodynamic diameter (Hd), polydispersity index (PDI), ζ-potential (ζ), pH, and conductivity of the empty and vitamin E-loaded nanoemulsions, after 24 h of preparation at different oil ratios, are summarized in Table 2.

**Table 2:** Nanoemulsion Properties (*n* = 3)^a^

**sample**	**Hd** (nm)	**PDI**	**ζ** (mV)	**pH**	**conductivity** (μS cm^**–1**^ **)**
**V.E**3:0	107.67 ± 7.57^a^	0.24 ± 0.01^c^	–37.4 ± 0.80c	6.14 ± 0.03^a^	753.30 ± 14.90^a^
**V.E**2:1	114.26 ± 6.51^a^	0.24 ± 0.01^b,c^	–49.0 ± 2.00^b,c^	5.84 ± 0.24^a^	806.10 ± 4.90^b,c^
**V.E**1:2	113.24 ± 3.25^a^	0.21 ± 0.01^a^	–44.6 ± 0.60^b^	5.90 ± 0.30^a^	822.10 ± 37.70^c^
**V.E**0:3	134.38 ± 3.15^b^	0.22 ± 0.01^a,b^	–45.3 ± 0.90^a^	5.90 ± 0.10^a^	798.10 ± 21.70^b^

a Means followed by different lowercase letters are significantly different (*p* < 0.05). Equal letters indicate no significant difference. (V.E = Vitamin E; Hd = hydrodynamic diameter; PDI = polydispersity index; ζ = ζ-potential.)

The data in Table 2 reveal distinct trends in nanoemulsion properties as the coconut oil-to-squalane ratio changes. Notably, the hydrodynamic diameter (Hd) increases with a higher squalane content, from 107.67 ± 7.57 nm at a 3:0 ratio (pure coconut oil) to 134.38 ± 3.15 nm at a 0:3 ratio (pure squalane). This trend can be attributed to the higher viscosity and larger molecular structure of squalane compared to coconut oil, which is rich in medium-chain fatty acids like lauric acid (Supporting Information Table S1). Squalane’s higher viscosity likely increases the resistance to droplet breakup during emulsification, resulting in larger droplets, as reported in similar systems.^52,53^

Conversely, coconut oil’s lower viscosity and smaller fatty acid chains facilitate smaller droplet formation, contributing to the smaller Hd at higher coconut oil ratios. The polydispersity index (PDI) remains relatively low (0.21–0.24), indicating uniform droplet size distributions across all ratios, with the lowest PDI (0.21 ± 0.01) observed at the 1:2 ratio, suggesting an optimal balance of the two oils for emulsion homogeneity. The ζ-potential (ζ) becomes more negative with increasing squalane content, from −37.4 ± 0.80 mV (3:0) to −45.3 ± 0.90 mV (0:3), reflecting enhanced electrostatic repulsion and stability due to squalane’s nonpolar nature, which may reduce interfacial tension in the presence of surfactants.^53^

The pH remains stable (5.84 to 6.14), suitable for topical applications, while conductivity increases slightly with higher squalane content (753.30 to 822.10 μS cm^–1^), possibly due to changes in the oil–water interface dynamics. Upon incorporation of vitamin E, a reduction in Hd was observed across all ratios, likely due to its methyl groups lowering interfacial tension and enhancing compatibility with the oil phase.^53^ This reduction in droplet size improves kinetic stability by inhibiting Ostwald ripening, a phenomenon counteracted by the mixing entropy of vitamin E in the oil phase.^52,53^

Upon incorporation of vitamin E, a reduction in the hydrodynamic diameter was observed across all oil ratios. For example, at the 3:0 ratio (pure coconut oil), the Hd decreased from 120.45 ± 8.12 nm for the empty nanoemulsion to 107.67 ± 7.57 nm for the vitamin E-loaded nanoemulsion, with similar reductions observed at other ratios (e.g., from 140.22 ± 4.10 to 134.38 ± 3.15 nm at the 0:3 ratio). This reduction may be attributed to a decrease in interfacial tension due to the presence of methyl groups in vitamin E, as well as an enhanced compatibility between vitamin E and the oil phase, particularly coconut oil’s lauric acid-rich composition, thereby promoting better organization of the system.^53^ This phenomenon was also noted by Ozturk et al.,^54^ who demonstrated that the addition of a poorly water-soluble active compound such as vitamin E during nanoemulsion formation could inhibit Ostwald ripening through the effects of mixing entropy, which counteracts the growth of nanoemulsion droplets (compositional ripening).

Additionally, the inclusion of vitamin E in systems utilizing natural surfactants, such as Quillaja and lecithin, resulted in a decrease in the hydrodynamic diameter. However, as noted by Ozturk et al.,^54^ an optimal oil phase concentration is necessary, as excessive oil content can increase the viscosity of the oil phase, thereby impeding homogenization efficiency and leading to larger droplet sizes. This effect is particularly relevant in the context of the coconut oil-to-squalane ratio, where squalane has a higher viscosity compared to coconut oil (Supporting Information Table S1) contributes to the observed increase in hydrodynamic diameter at higher squalane ratios (Table 2). Furthermore, they have reported that the viscosity of the oil phase, influenced by its composition and concentration, directly affects the emulsification process, with more viscous oils requiring greater energy input to achieve smaller droplet sizes.^54^ This viscosity-driven effect explains the larger droplet sizes observed at the 0:3 ratio (pure squalane) compared to the 3:0 ratio (pure coconut oil), highlighting the importance of optimizing the oil phase composition to balance the viscosity and emulsion stability.

The hydrodynamic diameter of the nanoemulsions is influenced by the composition of the system and the technique used during preparation. Generally, increasing the stirring speed tends to reduce the droplet size significantly; however, for certain emulsifiers (such as biopolymers), prolonged emulsification times may hinder the formation process.^52^ The characteristics of the nanoemulsion, such as the appearance and texture, are directly correlated with the droplet size. It has been reported that nanoemulsions with droplet sizes below 200 nm exhibit an improved kinetic stability and prolonged shelf life. Nevertheless, long-term storage can still lead to phenomena of instability.^53^

The stability of the nanoemulsions was monitored for 28 days, with additional evaluations performed at 60 and 90 days to assess the long-term performance under storage at 25 ± 1 °C in the dark.

Monitoring of the hydrodynamic diameter, PDI, ζ-potential, pH, and conductivity over 90 days is summarized in Table 3. Nanoemulsions containing only squalane exhibited larger hydrodynamic diameters than the others, in both unloaded and vitamin E-loaded systems, likely due to the physicochemical characteristics of the oils. Squalane consists of a 30-carbon chain,^55^ making it a relatively large molecule, in contrast to coconut oil, which comprises a mixture of medium-chain triglycerides.^56^ This structural difference may favor the formation of nanoemulsions with smaller droplet sizes in systems containing coconut oil compared with those formulated solely with squalane.

**Table 3:** Stability Parameters of Nanoemulsions at 1, 7, 14, 28, 60, and 90 Days^a^

**time** (days)	V.E 0:3	V.E 1:2	V.E 2:1	V.E 3:0
**Hd**				
1	134.38 ± 3.15^a^	113.24 ± 3.25^a^	114.26 ± 6.51^a^	107.67 ± 6.18^a^
7	133.00 ± 1.91^a^	110.96 ± 3.51^a^	105.00 ± 7.64	111.74 ± 11.24^a^
14	153.55 ± 6.51^b^	113.17 ± 3.05^a^	110.96 ± 15.71^a^	113.23 ± 5.81^a^
28	147.23 ± 1.40^a,b^	113.88 ± 3.09^a,b^	108.84 ± 10.82^a^	115.41 ± 14.22^a^
60	151.47 ± 1.20^b^	132.27 ± 9.82^c^	118.26 ± 5.57^a,b^	151.38 ± 0.60^b^
90	141.90 ± 1.38^a,b^	126.70 ± 0.25^b,c^	148.00 ± 0.26^b^	146.80 ± 0.24^b^
**PDI**				
1	0.22 ± 0.01^a,b^	0.21 ± 0.01^a^	0.24 ± 0.01^a^	0.24 ± 0.01^a^
7	0.21 ± 0.01^a^	0.21 ± 0.01^a^	0.21 ± 0.01^a^	0.25 ± 0.01^a^
14	0.25 ± 0.01	0.21 ± 0.01^a^	0.24 ± 0.02^a^	0.24 ± 0.01^a^
28	0.24 ± 0.01^b,c^	0.21 ± 0.01^a^	0.23 ± 0.01^a^	0.25 ± 0.01^a^
60	0.25 ± 0.01^c^	0.25 ± 0.01^b^	0.24 ± 0.03^a^	0.26 ± 0.02^a^
90	0.23 ± 0.01^a,b,c^	0.25 ± 0.01^b^	0.24 ± 0.02^a^	0.25 ± 0.01^a^
**ζ** (mV)				
1	–50.9 ± 1.3^c^	–46.0 ± 0.6^c^	–49.0 ± 1.9^d^	–36.5 ± 0.7^a^
7	–45.3 ± 0.8^b^	–44.6 ± 0.6^c^	–42.8 ± 3.1^c,d^	–33.7 ± 2.9^a^
14	–43.3 ± 0.4^a,b^	–41.1 ± 1.1^b^	–40.6 ± 4.5^c,d^	–40.6 ± 3.8^a^
28	–42.3 ± 1.4^a,b^	–38.8 ± 2.3^b^	–38.5 ± 7.1^b,c^	–38.4 ± 2.0^a^
60	–38.95 ± 4.2^a^	–34.1 ± 1.6^a^	–33.3 ± 3.2^a^	–34.3 ± 1.7^a^
90	–43.3 ± 0.7^a,b^	–35.1 ± 1.0^a^	–35.2 ± 0.8^a,b^	–40.9 ± 4.6^a^
**pH**				
1	5.90 ± 0.10^a^	5.90 ± 0.30^a^	5.84 ± 0.24^a^	6.14 ± 0.03^b^
7	5.41 ± 0.05^a^	5.42 ± 0.10^a^	5.34 ± 0.07^a^	4.57 ± 0.33^a^
14	5.81 ± 0.05^a^	5.74 ± 0.13^a^	5.77 ± 0.12^a^	4.94 ± 0.19^a^
28	5.87 ± 1.36^a^	5.36 ± 0.32^a^	5.79 ± 1.08^a^	5.01 ± 0.28^a,b^
60	5.50 ± 0.23^a^	5.25 ± 0.50^a^	5.81 ± 0.23^a^	5.00 ± 0.15^a,b^
90	5.21 ± 0.09^a^	5.06 ± 1.12^a^	5.60 ± 0.11^a^	4.95 ± 0.85^a^
**Conductivity** (μS cm^**–1**^ **)**				
1	798.1 ± 21.7^a^	822.1 ± 37.7^a^	806.1 ± 4.9^a^	753.3 ± 14.9^a^
7	881.4 ± 44.8^b^	882.2 ± 25.5^b,c^	905.0 ± 50.7^b,c^	906.6 ± 31.6^c^
14	942.9 ± 22.7^c^	971.4 ± 33.4^d^	963.7 ± 90.4^c^	814.1 ± 36.5^b^
28	909.0 ± 18.3^b,c^	924.9 ± 22.2^c,d^	917.6 ± 47.9^a,b,c^	774.7 ± 54.2^a^
60	923.7 ± 4.1^b,c^	879.6 ± 6.1^b^	887.3 ± 5.2^b,c^	836.9 ± 6.0^b,c^
90	939.9 ± 15.7^b,c^	975.7 ± 9.8^d^	919.0 ± 19.7^b,c^	943.3 ± 11.1^c^

a Results express mean ± standard deviation (*n* = 3). Same letters in the same column indicate no significant difference by Tukey’s test (*p* < 0.05).

No significant variation in the mean hydrodynamic diameter was observed over time in any of the formulations, either unloaded or vitamin-loaded. These results suggest that storage time did not affect the system stability. Typically, prolonged storage may influence nanoemulsion size due to destabilization processes, as the system tends to return to a thermodynamically stable state. In most cases, an increase in droplet size results from coalescence or Ostwald ripening, or from reduced electrostatic repulsion between droplets associated with pH variation.^57^ The absence of such changes after 90 days indicates that the systems remained stable throughout the storage period.

The PDI values demonstrated a uniform droplet size distribution, remaining between 0.21 and 0.28 for most formulations during the 90-day evaluation. According to the literature, PDI values below 0.30 indicate good monodispersity and stability.^58,59^ These results are directly related to the characteristics of the surfactants employed. Arbain et al.^60^ reported low-polydispersity nanoemulsions with smaller droplet sizes when combining Tween 80 and lecithin, due to their structural similarity, which enhances compatibility, produces a more homogeneous interface, and promotes better compound entrapment. The formation of nanoemulsions also depends on surfactant concentration, as higher concentrations tend to yield smaller particles due to greater availability of surfactant molecules to cover droplet surfaces during homogenization and faster adsorption at the oil–water interface.^61,62^

Regarding ζ-potential, all formulations exhibited high absolute values, indicating strong electrostatic repulsion between droplets. ζ-potential values were found to be dependent on storage time (*p* < 0.05). Similar results were reported by Paulo et al.^57^ for baru oil nanoemulsions stabilized with Tween 80, in which ζ-potential varied with storage time and pH, ranging from −50.5 to −39.4 mV. The authors attributed this variation to the deprotonation of oil components, which reduced surface charge.

ζ-potential is a key indicator of electrostatic stability: the higher the surface charge, the greater the stability of the nanoemulsion.^63^ Generally, values around ± 30 mV are considered adequate to ensure system stability,^59,64^ as high surface potentials produce strong repulsive forces between droplets, preventing flocculation and coalescence.^65^ This parameter depends on the type and concentration of surfactant used, serving as an important but not exclusive stability criterion.^66,67^ Negative ζ-potential values are often observed in systems containing lecithin or Tween surfactants, likely due to hydroxide ion adsorption at the oil–water interface arising from interactions between water molecules and the functional groups of Tween 80.^68^ The presence of negatively charged phospholipids at the droplet interface may further contribute to this effect. Although ζ-potential effectively reflects electrostatic interactions, nanoemulsion stability should be assessed comprehensively, considering hydrodynamic diameter, PDI, pH, conductivity, and environmental storage conditions.^69^

All formulations exhibited slight variations in conductivity over time; however, these changes did not significantly affect the stability. Alaayedi and Maraie^70^ reported similar behavior in lomustine nanoemulsions with varying water contents to determine the external phase nature. Because water has higher electrical conductivity than oil, the results confirmed the formation of O/W-type nanoemulsions with conductivity values ranging from 241 to 998 μS cm^–1^. Conductivity, like pH, can serve as an indicator of instability and can influence droplet size; a significant increase over time may be related to coalescence, whereas a decrease may indicate droplet aggregation.^71^ Therefore, notable shifts in conductivity may reflect possible instability reactions in the system, such as hydrolysis or oxidation, which release ions into the aqueous phase.

Human skin has a slightly acidic pH, typically ranging from 4.6 to 5.8. Maintaining this range is essential to preserve skin integrity, ensure bactericidal and fungicidal protection,^72^ and support enzymatic activity involved in ceramide synthesis.^73^ Significant deviations from this range may indicate microbial growth or chemical reactions that compromise product quality.^71^ In this study, all nanoemulsions maintained pH values within the range suitable for the dermal application. Moreover, pH serves as an indicator of chemical stability, as variations may signal degradation reactions. Nanoemulsions formulated with vegetable oils tend to exhibit a slight pH reduction over time due to hydrolysis of fatty acid esters.^74^ Sun et al.^75^ observed increased creaming in flaxseed oil emulsions under extremely acidic or alkaline conditions. This aggregation phenomenon generally occurs when attractive forces (van der Waals and hydrophobic) exceed repulsive ones (electrostatic and steric), particularly when electrostatic repulsion between droplets is weak under low or high pH.^76^ In the present study, pH values remained stable over time, further confirming the good stability of the nanoemulsion systems.

### 3.3 Morphological Analysis

The morphology of the nanoemulsions was evaluated by using scanning electron microscopy (SEM) to provide insights into their structural characteristics. Figure 2 illustrates the SEM images of the vitamin E-loaded nanoemulsions with different coconut oil-to-squalane ratios (V.E 3:0, V.E 2:1, V.E 1:2, V.E 0:3).

**Figure 2:**
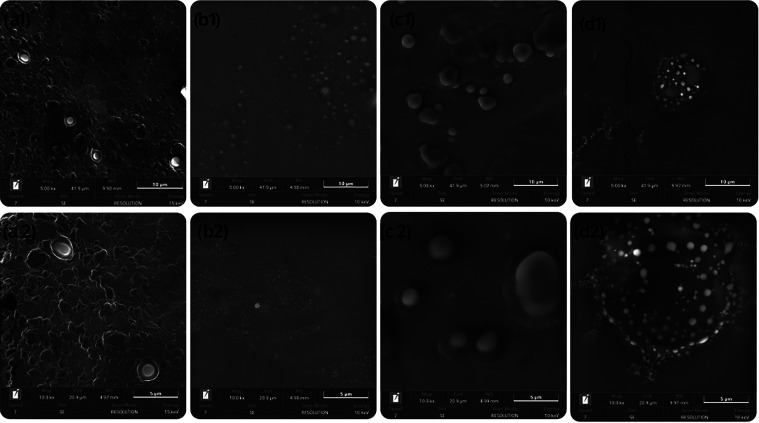
Micrographs of samples V.E 3:0 (a), V.E 2:1 (b), V.E 1:2 (c), and V.E 0:3 (d) at magnifications of 5.0 Kx (1) and 10.0 Kx (2), respectively.

The SEM images revealed spherical droplets with sizes consistent with the dynamic light scattering (DLS) measurements reported in Table 2, ranging from approximately 107.67 ± 7.57 (V.E 3:0) to 134.38 ± 3.15 nm (V.E 0:3). The V.E 0:3 formulation (pure squalane) exhibited slightly larger and more uniform droplets compared to the coconut oil-rich formulations, likely due to squalane’s higher viscosity and its influence on droplet formation during emulsification. The V.E 3:0 formulation (pure coconut oil) showed smaller, more tightly packed droplets, consistent with the lower viscosity of coconut oil’s medium-chain fatty acids, which facilitate finer droplet breakup during high shear homogenization. The V.E 1:2 and V.E 2:1 formulations displayed intermediate morphologies, with droplet sizes and distributions reflecting the balance of the two oils in the system.^77–81^

However, a critical limitation of the SEM analysis is the sample preparation method, which involves lyophilization and gold coating to ensure sample stability and conductivity under high-vacuum conditions. Lyophilization, which removes water from the nanoemulsions, may induce droplet aggregation, coalescence, or structural collapse, potentially altering the native morphology of the nanoemulsions in their aqueous solution state. Additionally, gold coating, while necessary for SEM imaging, may obscure fine surface details or introduce artifacts that do not reflect the true structure of the nanoemulsion droplets. Consequently, the SEM images provide valuable insights into the morphology of the dried nanoemulsions but may not fully represent their structure and behavior in solution, where they are intended for topical application. To address this limitation, future studies should incorporate complementary techniques such as transmission electron microscopy (TEM) or cryoelectron microscopy (cryo-EM), which allow visualization of nanoemulsions in their hydrated state without the need for drying or coating, thereby minimizing artifacts and providing a more accurate representation of their solution-phase morphology. These techniques would enable a more comprehensive understanding of the nanoemulsions’ structural integrity and behavior under conditions relevant to their practical use.^77–81^

Despite these limitations, the SEM analysis confirms the formation of nanoscale, spherical droplets across all formulations, supporting the DLS data and indicating that the coconut oil-to-squalane ratio influences droplet morphology. These findings provide a foundation for understanding the structural characteristics of the nanoemulsions and their potential for effective vitamin E delivery.

### 3.4 Vitamin E Release Kinetics by Dialysis

The release kinetics of vitamin E were evaluated through dialysis assays. It was observed that certain formulations did not exhibit a controlled release profile. Specifically, the 2:1 V.E nanoemulsion showed detectable release only after 24 h, reaching a concentration of 8.82 μg mL^–1^. Similarly, no detectable release of vitamin E was observed for the V.E 3:0 formulation during the 24 h dialysis assay, likely due to the strong interaction between vitamin E and the coconut oil-rich matrix, which impeded diffusion across the dialysis membrane. This behavior is likely associated with the higher concentration of coconut oil in this formulation, which may have hindered the diffusion of the active compound.

Nanoemulsions demonstrating a controlled release profile were further analyzed using classical mathematical models, including zero-order, first-order, Higuchi,^40^ and Korsmeyer–Peppas^41^ models, as illustrated in Figure 3. Fitting parameters of the release kinetic models based on the dialysis assay can be visualized in Table 4.

**Figure 3:**
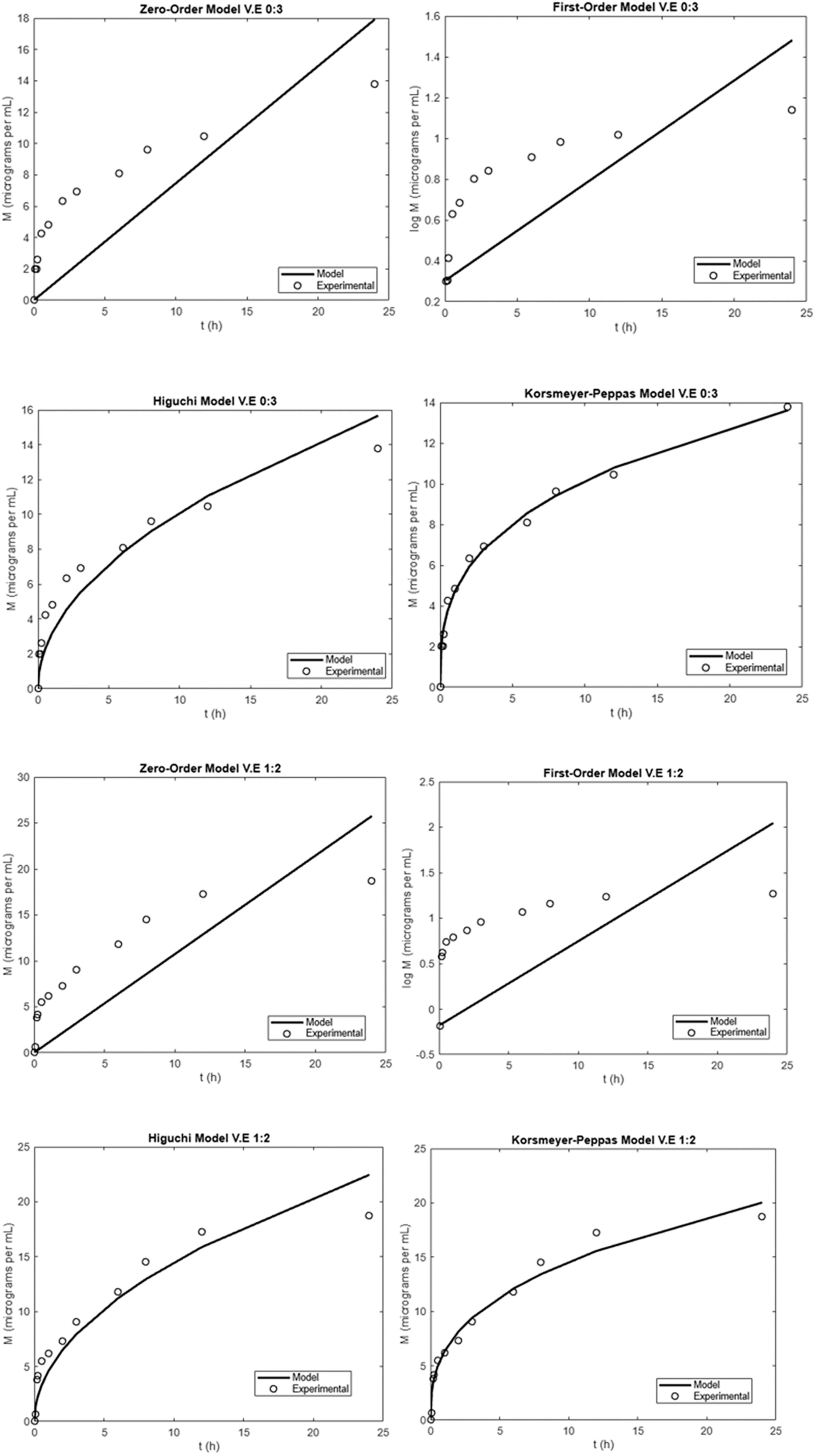
Mathematical modeling of vitamin E release kinetics from nanoemulsions using zero-order, first-order, Higuchi, and Korsmeyer-Peppas models.

**Table 4:** Fitting Parameters of the Release Kinetic Models Based on the Dialysis Assay, Indicating Model Performance through *R* ^2^ and RMSE Values, Release Rate Constant (*k*), and Release Exponent (*n*) for the Korsmeyer-Peppas Model

**sample**	**model**	***R*** ^**2**^	**RMSE**	**constant** (*k*)	***n***
V.E 1:2	zero-order	0.348	47.605	1.073	
	first-order	–2.394	0.0476	0.214	
	Higuchi	0.914	17.258	4.582	
	Korsmeyer-Peppas	0.974	0.9514	0.337	0.363
V.E 0:3	zero-order	0.253	33.699	0.747	
	firs- order	0.165	0.1721	0.153	
	Higuchi	0.896	12.566	3.197	
	Korsmeyer-Peppas	0.993	0.3319	0.341	0.335

The release data for the 1:2 and 0:3 V/E samples exhibited a good fit to the Korsmeyer–Peppas model, with coefficients of determination (*R* ^2^) of 0.974 and 0.993, respectively. Additionally, the root-mean-square error (RMSE) values supported the adequacy of the model fit.

The V.E 0:3 formulation, containing only squalane as the oil phase, exhibited a faster and more sustained release profile compared to V.E 1:2, which has a 1:2 ratio of coconut oil to squalane. Specifically, V.E 0:3 achieved a higher cumulative release of vitamin E over the 24 h period, with a release rate constant (*k*) of 0.341 and a release exponent (*n*) of 0.335, indicating Fickian diffusion. In contrast, V.E 1:2 showed a slightly lower release rate (*k* = 0.337, *n* = 0.363), suggesting a marginally slower diffusion process. This difference can be attributed to the higher squalane content in V.E 0:3, which likely reduces the viscosity of the oil phase and enhances the compatibility of vitamin E with the lipid matrix, thereby facilitating faster diffusion across the dialysis membrane. The presence of coconut oil in V.E 1:2, with its medium-chain fatty acids, such as lauric acid, may increase the viscosity and strengthen the interaction with vitamin E, leading to a more gradual release. These findings highlight the critical role of the oil phase composition in modulating the release kinetics of vitamin E from the nanoemulsions.

The Korsmeyer–Peppas model, an extension of the Higuchi model, is widely employed to describe drug release from polymeric systems. It provides insights into the mechanisms involved in the release process from matrices undergoing erosion and/or dissolution.^41^ In this model, the exponent “n” characterizes the release mechanism. For spherical systems like nanoemulsions, an n value of approximately 0.43 indicates Fickian diffusion (Case I), where release is governed purely by diffusion, while n values between 0.43 and 0.85 suggest anomalous (non-Fickian) transport, involving both diffusion and matrix relaxation or swelling. Values of n below 0.43, as observed in this study (0.335 for V.E 0:3 and 0.363 for V.E 1:2), also indicate anomalous transport, likely due to a combination of diffusion and structural changes in the nanoemulsion matrix, such as swelling or reorganization of the oil–water interface, influenced by the surfactant and oil phase composition. These n-values suggest that the release of vitamin E is not solely diffusion-controlled but is also affected by the dynamic behavior of the nanoemulsion droplets, potentially due to the interaction between vitamin E and the squalane or coconut oil matrix. For instance, the slightly higher n-value for V.E 1:2 (0.363) compared to V.E 0:3 (0.335) may reflect a greater contribution of matrix relaxation in the presence of coconut oil, which has a more complex fatty acid profile that could influence droplet swelling. These findings indicate that the release mechanism is complex and warrants further investigation to elucidate the specific contributions of diffusion and matrix dynamics in these systems.^82,83^

Several factors can influence the release behavior of actives from nanoemulsions. In passive release scenarios, diffusion typically follows Fick’s First Law. Highly lipophilic compounds, such as vitamin E, may require the lipidic barrier of the formulation to be released. However, nanoemulsions possess a high interfacial area, which is expected to facilitate faster release rates.^83^ Additionally, the small droplet size in nanoemulsions can enhance the diffusion dynamics. Conversely, the incorporation of vehicles such as gels^84^ or polymeric coatings may slow down release. Surfactants also play a key role by forming compact interfacial layers, which can modulate or even control the release kinetics of encapsulated compounds.^83^

### 3.5 Skin Permeation Studies

In vitro release and skin permeation studies are essential for evaluating the efficacy of topical formulations.^30,85^ Among the available methodologies, the Franz diffusion cell system remains the most widely adopted due to its advantages, including low sample requirements, cost-effectiveness, and the ability to simulate the diffusion of active compounds through the skin barrier.^86^ In the present study, nanoemulsions were subjected to skin permeation testing by using a Franz cell system, as illustrated in Figure 4.

**Figure 4:**
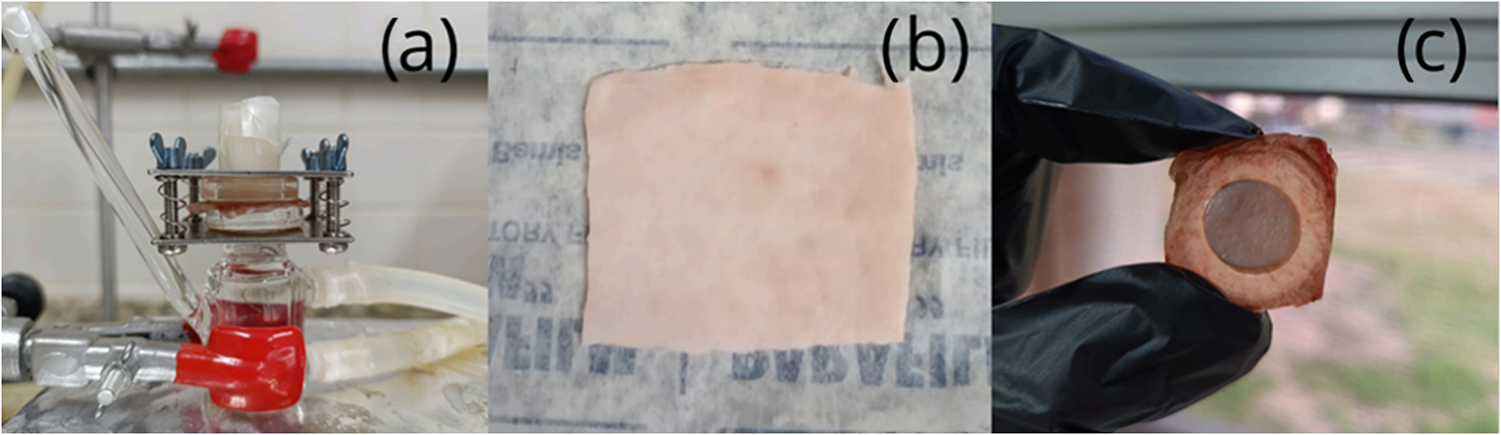
Mechanism of skin permeation using the Franz diffusion Cell. (a) Assembled system; (b) skin before the test; (c) skin after the test.

Vitamin E solubilized in sweet almond oil was used as a control to assess its release behavior outside of the nanoemulsion system. Vitamin E was detected in the receptor compartment for all samples; however, significant differences in the amount released over 24 h were observed, as detailed in Table 5. Notably, nanoemulsions formulated solely with squalane exhibited the highest release of vitamin E, as demonstrated by the V.E 0:3 formulation (164.7 μg cm^–2^). Likewise, V.E 1:2, which contains a higher proportion of squalane relative to coconut oil, also showed enhanced permeation (70.2 μg cm^–2^) compared with other formulations.

**Table 5:** Vitamin E Concentrations in the Receptor Compartment and Skin, Percentage of Vitamin E Released, Steady-State Flux (Jss), and Permeability Coefficient (Kp)^a^

**sample**	**receiver compartment** (μg cm^**–2**^ **)**	**amount retained in the skin** (μg cm^**–2**^ **)**	**vitamin E released** (%)	**Jss** (μg cm^**2**^ **h** ^**–1**^ **)**	**Kp (×10** ^**–3**^ cm h^**–1**^ **)**
V.E 0:3	164.71 ± 10.06^c^	873.3 ± 3.2^c^	1.3 ± 0.6^a,b^	35.1 ± 1.4^c^	1.8 ± 0.6^a^
V.E 1:2	70.23 ± 7.52^b^	941.7 ± 4.1^d^	1.3 ± 1.2^a,b^	21.0 ± 2.7^b^	1.1 ± 0.2^a^
V.E 2:1	6.30 ± 6.12^a^	2000.0 ± 12.4^e^	2.5 ± 0.9^c^	0.1 ± 0.2^a^	0.1 ± 0.1^a^
V.E 3:0	11.72 ± 12.41^a^	510.4 ± 9.7^b^	0.7 ± 0.1^a^	0.1 ± 0.2^a^	01 ± 03^a^
control	57.10 ± 4.19^b^	158.0 ± 8.1^a^	0.2 ± 0.1^a^	47.0 ± 8.8^d^	2.3 ± 0.8^a^

a Values are expressed as mean ± SD (*n* = 3). Values followed by different lowercase letters are significantly different (*p* < 0.05). Equal letters indicate no significant difference.

The superior permeation observed with squalane-rich formulations (V.E 0:3 and V.E 1:2) can be attributed to squalane’s unique physicochemical properties and its interaction with the skin’s lipid matrix. Squalane, a saturated hydrocarbon derived from squalene, is a nonpolar, lipophilic molecule with a branched structure that closely mimics the lipid composition of the stratum corneum.^20,21^ This structural similarity facilitates its integration into the intercellular lipid lamellae of the stratum corneum, temporarily disrupting its ordered structure and enhancing its fluidity. This disruption lowers the diffusional resistance of the skin barrier, allowing for improved partitioning and diffusion of lipophilic compounds like vitamin E (log*P* ≈ 10) into and through the stratum corneum.^29^ The branched molecular structure of squalane further contributes to its penetration-enhancing effect by reducing the packing density of skin lipids, creating transient pathways for vitamin E diffusion. In contrast, coconut oil, rich in medium-chain fatty acids such as lauric acid (log*P* ≈ 4.6), has a more polar character and forms stronger intermolecular interactions with vitamin E, potentially reducing its release from the nanoemulsion matrix and favoring retention within the skin rather than transdermal permeation.^21,46^ This structure–activity relationship explains the observed trend where increasing squalane content correlates with higher vitamin E permeation (Table 5), as squalane’s nonpolar nature and branched structure enhance its ability to act as a penetration enhancer compared to the more polar and linear fatty acids in coconut oil.

Comparative analysis with prior studies further supports these findings. Kong et al.^29^ reported a cumulative vitamin E permeation of 8.29 ± 2.88 μg mL^–1^ over 24 h from nanoemulsions using methylene chloride as the oil phase and a Tween 80/Span 20 surfactant system, significantly lower than the 164.71 ± 10.06 μg cm^–2^ achieved by V.E 0:3 in this study. This disparity can be attributed to squalane’s superior penetration-enhancing properties compared to methylene chloride, which is a volatile solvent with limited compatibility with the skin’s lipid matrix. Schreiner et al.^30^ achieved vitamin E skin retentions of 11–21 μg cm^–2^ using a saponin-stabilized nanocream, which, while effective for retention, is notably lower than the retention (873.3 ± 3.2 μg cm^–2^ for V.E 0:3) and permeation observed here. The higher retention and permeation in our study likely result from squalane’s ability to both integrate into the stratum corneum and facilitate vitamin E diffusion, whereas saponins primarily stabilize the emulsion without significantly enhancing penetration.^30^ Similarly, Wilbert-Lampen et al.^87^ reported a stratum corneum retention of 12 μg cm^–2^ for a 2% vitamin E cream, highlighting the limited permeation of traditional formulations compared to squalane-based nanoemulsions. These comparisons underscore the critical role of squalane’s molecular structure in enhancing both retention and permeation, distinguishing our formulations from those relying on less effective oil phases or stabilizers.

The structure–activity relationship is further elucidated by considering the physicochemical properties of the oil phase. Squalane’s high lipophilicity (log*P* ≈ 15) and branched structure allow it to interact favorably with the hydrophobic tails of stratum corneum lipids, promoting partitioning of vitamin E into the skin. In contrast, coconut oil’s medium-chain fatty acids, such as lauric acid, have lower log*P* values and form more cohesive interactions with vitamin E, leading to higher skin retention (e.g., 2000.0 ± 12.4 μg cm^–2^ for V.E 2:1) but reduced permeation (6.30 ± 6.12 μg cm^–2^). This is consistent with Oliveira et al.,^21^ who demonstrated that squalane in W/O emulsions extended polyphenol retention by reducing transdermal flux, suggesting that squalane’s effect depends on the emulsion type and active compound. In O/W nanoemulsions, as used here, squalane appears to balance retention and permeation, making it ideal for cosmetic applications where localized delivery to the stratum corneum and viable epidermis is desired.^88^ The Korsmeyer-Peppas release exponent (*n* ≈ 0.335–0.363, Table 4) further indicates anomalous transport, suggesting that squalane not only enhances diffusion but also modulates droplet dynamics, potentially through interfacial reorganization, which facilitates vitamin E release.^41^

In the present study, nanoemulsions formulated with higher proportions of coconut oil—specifically the V.E 2:1 and V.E 3:0 systems, exhibited significantly greater skin retention of vitamin E. The stratum corneum, being the primary barrier to percutaneous absorption, plays a crucial role in the delivery of cosmetic actives. For topical applications, it is generally desirable that active compounds penetrate and be retained within the stratum corneum and viable epidermis, without reaching systemic circulation.^30,88^ Accordingly, the observed results suggest that the nanoemulsions developed herein effectively promote the localized retention of vitamin E, in alignment with the goals of cosmetic formulations. Kong et al.^29^ also emphasized the importance of such retention, noting that the majority of the active compound should ideally remain in the skin rather than being systemically absorbed.

Regarding the final stages of in vitro dermal absorption assays, the evaluation methodology varies depending on the application type—finite or infinite dose. In finite dose studies, the focus is on quantifying the maximum average absorption and requiring complete recovery of the test substance. In contrast, infinite dose protocols prioritize the determination of steady-state flux and the calculation of the permeability coefficient (Kp), where full recovery is not essential; rather, the appearance of the compound in the receptor medium is the key parameter.^87,89,90^ In this study, both flux and permeability coefficient values were calculated and are reported in Table 5. The Kp value is a critical parameter in percutaneous absorption studies, serving as a predictive indicator of dermal penetration.^91^ The V.E 0:3 formulation exhibited a steady-state flux (Jss) of 35.1 ± 1.4 μg cm^–2^ h^–1^ and a permeability coefficient (Kp) of 1.8 ± 0.6 × 10^–3^ cm h^–1^, significantly higher than the coconut oil-rich V.E 3:0 (Jss = 0.1 ± 0.2 μg cm^–2^ h^–1^, Kp = 0.1 ± 0.3 × 10^–3^ cm h^–1^). These values reflect squalane’s ability to enhance vitamin E diffusion, likely due to its interaction with the stratum corneum lipids, as discussed above.

The release profiles of vitamin E from the tested nanoemulsion systems are illustrated in Figure 5. Notably, the V.E 0:3 formulation demonstrated significantly different release behavior. Vitamin E was first detected in the receptor medium 2 h after application, followed by a rapid increase in concentration during the subsequent hours. From the sixth hour onward, the release plateaued, indicating a sustained release phase. This profile aligns with squalane’s penetration-enhancing mechanism, which facilitates early diffusion followed by sustained delivery as vitamin E partitions into the skin.^92^ The V.E 1:2 formulation exhibited a delayed release profile during the initial 4 h, followed by a marked increase in the release rate up to the sixth hour, after which a plateau phase was observed, persisting until the conclusion of the assay. In contrast, the V.E 2:1 formulation demonstrated minimal release within the first hour, which remained relatively constant throughout the remainder of the experimental period, consistent with the stronger retention induced by coconut oil’s fatty acid composition.

**Figure 5:**
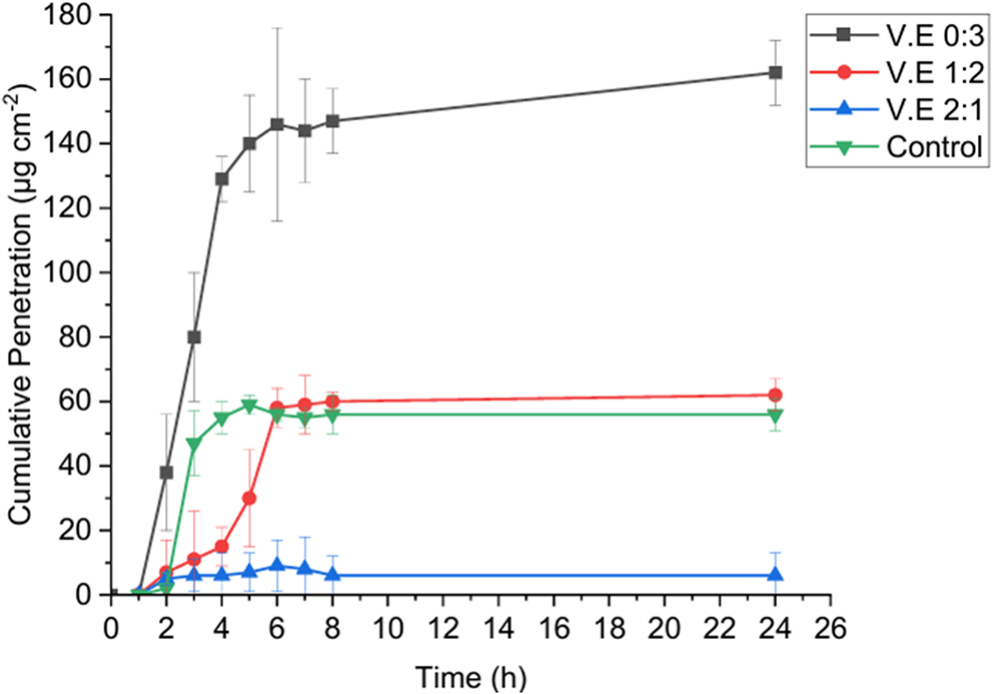
Cumulative permeation of vitamin E over time for all nanoemulsion formulations compared to that of a control sample (vitamin E in sweet almond oil).

According to Kong et al.,^29^ the transdermal penetration of lipophilic compounds in nanoemulsified systems is governed primarily by two factors: the presence of permeation enhancers, such as specific surfactants employed in the formulation, and the concentration of the donor phase. Both increasing the donor concentration and incorporating effective permeation enhancers have been shown to enhance the efficiency of percutaneous absorption. Lipophilic substances exhibit enhanced skin absorption due to their ability to interact with the lipid matrix of the stratum corneum via partitioning mechanisms. This effect is particularly pronounced for compounds with log*P* values ranging from −1 to 3.5. However, vitamin E’s high lipophilicity (log*P* ≈ 10) suggests that its permeation is more dependent on the carrier’s ability to disrupt the stratum corneum’s lipid barrier, as achieved by squalane.^92^ The surfactant system (soy lecithin and Tween 80) used in our formulations further supports this process by stabilizing the nanoemulsion and reducing interfacial tension, enhancing vitamin E’s partitioning into the skin.^53^

Additionally, skin permeability is modulated by the physicochemical properties of the active compound, particularly its molecular weight and size. Amphiphilic molecules with lower molecular masses (<150 Da) tend to penetrate the skin more efficiently.^21,92^

Vitamin E, with a molecular weight of approximately 430 Da, relies on the carrier system to overcome this limitation. Squalane’s ability to enhance permeation, as evidenced by the high Jss and Kp values for V.E 0:3, compensates for vitamin E’s relatively large molecular size, making it an effective carrier for lipophilic actives in topical applications.^92^ This structure–activity relationship highlights the importance of selecting an oil phase with optimal lipophilicity and structural compatibility with the skin’s lipid matrix to maximize delivery efficiency.

## 4 Conclusion

This study successfully demonstrated the effective formulation of O/W nanoemulsions utilizing squalane as a carrier for the delivery of vitamin E. The prepared nanoemulsions exhibited favorable physicochemical properties, including appropriate particle size (107.67 ± 7.57 to 134.38 ± 3.15 nm), polydispersity index (0.21–0.24), and ζ-potential (−37.4 ± 0.80 to −45.3 ± 0.90 mV), indicating good stability and potential for application in cosmetic or dermatological products. The optimization of agitation speed at 15,000 rpm, validated by 90-day stability data, ensured robust emulsion formation and long-term stability under controlled conditions. Extended stability testing over 90 days confirmed maintenance of these properties under dark storage at 25 °C, with high encapsulation efficiency (>99%) and robustness under accelerated tests (centrifugation, thermal stress).

In vitro release studies, conducted using dialysis, confirmed the potential for controlled release of vitamin E from the nanoemulsions, with the V.E 0:3 formulation (pure squalane) achieving a release rate constant of 0.341 h^–1^ (Korsmeyer-Peppas model, *R* ^2^ = 0.993). Furthermore, skin permeation studies using Franz diffusion cells and porcine ear skin revealed that squalane plays a crucial role in enhancing the permeation of vitamin E across the skin barrier, with V.E 0:3 demonstrating significantly higher permeation (164.71 ± 10.06 μg cm^–2^ over 24 h, *p* < 0.05) compared to coconut oil-rich formulations (e.g., V.E 3:0:11.72 ± 12.41 μg cm^–2^). The results indicate that squalane is a promising component for enhancing the delivery of vitamin E, offering potential benefits in topical formulations aimed at improving skin health and addressing dermatological conditions.

Despite these promising results, the study has notable limitations. The reliance on in vitro Franz cell assays, while valuable for assessing permeation and retention, does not fully replicate the dynamic physiological environment of human skin, including metabolic processes, immune responses, and blood flow, which may influence delivery outcomes. Furthermore, no cytotoxicity, skin irritation, or sensitization tests were conducted, which are critical for confirming the safety of these formulations for prolonged skin contact. Scaling up production may also pose challenges such as maintaining consistent droplet size and stability during large-scale emulsification, which requires further investigation.

To advance clinical translation, we recommend conducting in vivo studies in animal models (e.g., mice or pigs) to validate the efficacy of squalane-based nanoemulsions under physiological conditions. Human volunteer studies should be conducted to assess performance in real-world settings, focusing on antiaging efficacy and skin health outcomes. Cytotoxicity and skin irritation tests, adhering to OECD guidelines (e.g., OECD 431 for skin corrosion), are essential to ensure biocompatibility, particularly given squalane’s established safety profile in cosmetics. Additionally, formulation optimization for scalability, such as adjusting surfactant ratios or exploring alternative emulsification techniques (e.g., high-pressure homogenization), will be critical to meet regulatory standards for cosmetic products (e.g., FDA or EU Cosmetic Regulation requirements).

The commercial significance of these findings lies in the potential of squalane-based nanoemulsions to meet growing consumer demand for effective, sustainable, and plant-derived antiaging products. The enhanced permeation and retention of vitamin E (up to 164.71 and 873.3 μg cm^–2^, respectively) position these formulations as competitive candidates for premium cosmetic products targeting skin aging and oxidative stress. Their biocompatibility, leveraging squalane’s presence in human skin lipids, and synergy with coconut oil’s antibacterial properties offer additional therapeutic benefits, such as improved skin barrier function and protection against pathogens like *P. acnes*. These attributes align with market trends favoring multifunctional cosmeceuticals, with potential applications in dermatological treatments for conditions such as atopic dermatitis or acne. Future research should focus on long-term stability studies, in vivo validation, and regulatory compliance to facilitate commercialization, ensuring that these nanoemulsions can be integrated into high-value cosmetic and therapeutic products.

## Supplementary Material



## Data Availability

All data generated or analyzed during this study are included in this published article.

## Notes

Fatty acid profile of coconut oil (Supplementary Figure 1) and quantification of fatty acids present in coconut oil used as carrier, presented in milligrams per gram of total lipids (TL) (Supplementary Table 1) (PDF)
