# Supplementary material for: Effect of Squalane as a Carrier in O/W Nanoemulsions for Dermal Delivery of Vitamin E
Source: ACS Omega. 2025 Dec 3;10(49):60707–22. doi: 10.1021/acsomega.5c08561 (PMC12713435; doi:10.1021/acsomega.5c08561)
Supplement: Supplementary file 1 [file ao5c08561_si_001.pdf]

Effect of squalane as a carrier in O/W nanoemulsions for dermal delivery of vitamin E

Aniely Cristina de Souza[1], Caroline Casagrande Sipoli[1], Rafael Block Samulewski[1], Ana Caroline Raimundini Aranha[2], Rafael Oliveira Defendi[1], Rúbia Michele Suzuki[1]*

[1]Universidade Tecnológica Federal do Paraná, Postgraduate Program in Chemical Engineering (PPGEQ-AP), Marcilio Dias 635, CEP 86812-460, Apucarana, PR, Brazil

[2]Universidade Estadual de Maringá, Postgraduate Program in Chemical Engineering, Department of Chemical Engineering, Av. Colombo 5790, Bloco D90, CEP 87020-900 Maringá, PR, Brazil

*Corresponding author: Rafael Block Samulewski

E-mail address: samulewski@utfpr.edu.br

Supporting Information

Supporting information is available free of charge via the Internet at http://pubs.acs.org. The file includes Supplementary Figure 1, presenting the fatty acid profile of coconut oil, and Supplementary Table 1, which provides the quantification of fatty acids present in the coconut oil used as carrier, expressed in milligrams per gram of total lipids (TL).

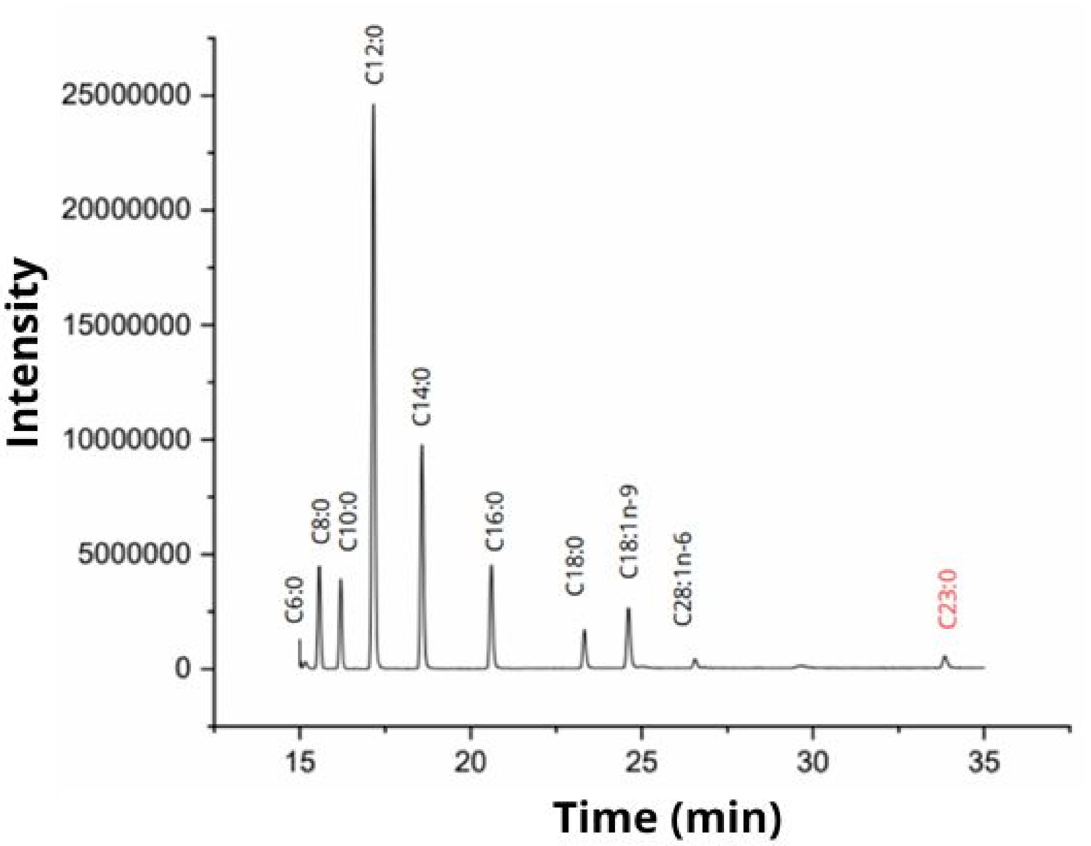

Supplementary Figure 1. Fatty Acid Profile of Coconut Oil.

Supplementary Table 1. Quantification of fatty acids present in coconut oil used as carrier, presented in milligrams per gram of total lipids (TL).

**Fatty acid**	**mg (g TL)$^{-1}$**
C6:0	5.51 ± 1.04
C8:0	162.28 ± 5.58
C10:0	138.79 ± 7.72
C12:0	845.55 ± 5.05
C14:0	410.85 ± 5.05
C16:0	197.13 ± 14.06
C18:0	72.42 ± 13.07
C18:1n-9	120.82 ± 1.52
C18:2n-6	10.46 ± 1.52
